# Hinge Region in DNA Packaging Terminase pUL15 of Herpes Simplex Virus: A Potential Allosteric Target for Antiviral Drugs

**DOI:** 10.3390/biom9100603

**Published:** 2019-10-12

**Authors:** Lana F. Thaljeh, J. Ainsley Rothschild, Misagh Naderi, Lyndon M. Coghill, Jeremy M. Brown, Michal Brylinski

**Affiliations:** 1 Department of Biological Sciences, Louisiana State University, Baton Rouge, LA 70803, USA; lthalj1@lsu.edu (L.F.T.); jroths2@lsu.edu (J.A.R.); mnader5@lsu.edu (M.N.); lcoghill@lsu.edu (L.M.C.); jembrown@lsu.edu (J.M.B.); 2 Center for Computation & Technology, Louisiana State University, Baton Rouge, LA 70803, USA

**Keywords:** herpesviruses, herpes simplex virus 1, HHV-1, DNA packaging terminase, pUL15, DNA packaging motor

## Abstract

Approximately 80% of adults are infected with a member of the herpesviridae family. Herpesviruses establish life-long latent infections within neurons, which may reactivate into lytic infections due to stress or immune suppression. There are nine human herpesviruses (HHV) posing health concerns from benign conditions to life threatening encephalitis, including cancers associated with viral infections. The current treatment options for most HHV conditions mainly include several nucleoside and nucleotide analogs targeting viral DNA polymerase. Although these drugs help manage infections, their common mechanism of action may lead to the development of drug resistance, which is particularly devastating in immunocompromised patients. Therefore, new classes of drugs directed against novel targets in HHVs are necessary to alleviate this issue. We analyzed the conservation rates of all proteins in herpes simplex virus 1 (HHV-1), a representative of the HHV family and one of the most common viruses infecting the human population. Furthermore, we generated a full-length structure model of the most conserved HHV-1 protein, the DNA packaging terminase pUL15. A series of computational analyses were performed on the model to identify ATP and DNA binding sites and characterize the dynamics of the protein. Our study indicates that proteins involved in HHV-1 DNA packaging and cleavage are amongst the most conserved gene products of HHVs. Since the packaging protein pUL15 is the most conserved among all HHV-1 gene products, the virus will have a lower chance of developing resistance to small molecules targeting pUL15. A subsequent analysis of the structure of pUL15 revealed distinct ATP and DNA binding domains and the elastic network model identifies a functionally important hinge region between the two domains of pUL15. The atomic information on the active and allosteric sites in the ATP- and DNA-bound model of pUL15 presented in this study can inform the structure-based drug discovery of a new class of drugs to treat a wide range of HHVs.

## 1. Introduction

Approximately 80% of the world population are infected with a form of herpes viruses [1]. Herpes viruses remain for the life of the host and can establish latency, a non-productive state that allows the virus to go undetected and unaffected by antiviral drugs [2,3]. There are nine herpes viruses that infect humans and their effects can range from easily manageable to life threatening [4]. Herpes simplex viruses 1 and 2 (HHV-1 and HHV-2) are associated with blisters on the lips and genitals, respectively, whereas HHV-6 and HHV-7 cause Roseola in infants [5]. Human cytomegalovirus (CMV or HHV-5) is linked to lethal diseases involving the lungs, gastrointestinal tract, liver, retina, and central nervous system in immune compromised individuals, and is the leading cause of post-transplant infection [6,7]. Congenital CMV results in an estimated 8000 cases of permanent neurological disabilities a year, affecting over 90% of infants who survive the initial infection [8,9,10]. Chicken pox and shingles, caused by varicella-zoster virus (VZV), are manageable in immunocompetent people, but can become deadly in immunocompromised persons [4,11]. Herpes viruses also are associated with several cancers. Epstein–Barr virus (EBV) causes Burkett’s lymphoma, nasopharyngeal carcinoma, and Hodgkin’s disease, and has been detected in approximately 70% of advance breast cancer tumors [12,13]. Kaposi’s sarcoma-associated herpesvirus (KSHV) can result in body cavity-based lymphoma, and Kaposi’s sarcoma, the most common malignancy present in HIV-1 patients [14,15]. The pervasiveness and deleterious effects of herpes viruses provides incentive for new approaches to antiviral drugs.

Widely used as a model to study herpesvirus replication, gene expression, and pathogenesis, the prototype virus of the family herpesviridae, HHV-1, has a linear double-stranded DNA genome of approximately 152 kbp. Several HHV-1 genomes have been sequenced to date, including strain 17 [16], strain KOS [17], and strain McKrae [18]. Similar to other members of the herpesvirus family, the HHV-1 genome consists of two unique regions, unique long (UL) and unique short (US), flanked by terminal inverted repeats [19]. The UL sequence contains 107,943 residues and has a base composition of 66.9% GC [16]. Among 56 genes identified in the UL region, accounting for most of the DNA sequence, the UL1, UL43, US7, UL23 and UL49 genes accumulated the majority of non-synonymous mutations across the genome [20]. The UL23 gene, for instance, has one of the highest genetic variabilities at 35.2%, confirming its role in the development of drug resistance [21]. The assembly of accurate, full-length HHV-1 genomes is critical to identify the genetic determinants of drug resistance, virulence, and pathogenesis.

The most common antiviral strategy against herpes viruses is based on nucleoside analogs targeting the viral DNA polymerase and thymidine kinase. While these drugs are effective, many of them are associated with significant toxicities, poor bioavailability, and resistance in immunocompromised persons [22]. The nucleoside analogues acyclovir and ganciclovir are the standard therapy for HHV-1 and CMV, respectively [22,23,24]. These compounds target HHV-1 pUL30, the viral DNA polymerase, and HHV-1 pUL23, the viral thymidine kinase. Regarding CMV, mutations in the viral kinase pUL97 and polymerase pUL54 mediate resistance to ganciclovir and valganciclovir [25]. The prevalence of resistance against acyclovir is 5% in immunocompromised persons and as high as 30% in allogeneic bone marrow transplant patients [26], whereas the incidence of resistance to ganciclovir is 5–10% in organ transplant recipients [27] and 40–50% in patients receiving repeated treatments or prolonged prophylaxis [25]. HHV-1 strains that are resistant to acyclovir are typically cross-resistant to thymidine kinase-dependent drugs such as penciclovir and famciclovir. They also may be cross-resistant to polymerase dependent drugs, foscarnet or cidofovir [26]. Ganciclovir-resistant strains also have shown cross resistance to second-line treatments foscarnet and cidofovir [28]. Targeting the DNA polymerase and thymidine kinase, while widely used, is not the only option for herpesvirus antivirals.

Due to the resistance, toxicities, and other adverse side effects of nucleoside analogs, new targets to treat herpes viruses are being perused. The viral DNA packaging motor, namely its large terminase subunit, has become a promising target for potential herpes antivirals [29]. The HHV-1 terminase is composed of three subunits which play several important roles in the packaging process, including identifying the viral concatemeric DNA, as well as endonuclease and ATPase activity. This provides several critical mechanisms that could be interrupted, provided a feasible drug binding site. Inhibiting the viral terminase or other components of the viral DNA packaging motor is expected to be more effective and has less target-related toxicity than nucleoside analogs. This is due to the fact that the molecular functions of the DNA packaging motor, capsid formation, DNA cleavage, and packaging of DNA into capsids, are virus-specific and not found in mammalian cells [30,31]. Current CMV terminase inhibitors include benzimidazoles and the 3,4-dihydro-quinazoline-4-yl-acetic acid derivative, letermovir [32].

The capsid needs to be filled with the appropriate DNA for a viable viral particle to be formed. Replication of the HHV genome first creates a concatemer and, subsequently, the packaging motor identifies the genetic sequence and begins packaging. At a certain point in the packaging process it must cleave the DNA in the proper location so only a monomeric strand of DNA remains in the capsid [33]. The first antiviral agents to inhibit the cleavage of concatenated DNA into monomeric genomes, through inhibiting the terminase, were 2,5,6-trichloro-1-β-D-ribofuranosyl benzimidazole (TCRB) and 2-bromo-5,6-dichloro-1-(β-D-ribofuranosyl) benzimidazole (BDCRB). These compounds are suspected to affect CMV proteins encoded by genes UL56 coding the small terminase subunit and UL89 coding the large terminase subunit, the key components of the packaging motor. Mutations in UL56 result in resistance to TCRB, whereas mutations in UL89 cause resistance to both TCRB and BDCRB [34,35]. The high resistance caused by mutations in UL89 were mapped to amino acid substitutions D344E and A355T [35]. Since there are no acidic residues corresponding to D344 of CMV in the HHV and VZM UL89 homologs, BDCRB and TCRB are unlikely to interact with HHV and VZV. This accords with in vitro results showing that benzimidazole ribonucleosides have little to no effect against HHV, VZV, HHV-6, and HHV-8 [30]. Along with the specific nature of these compounds, they also are metabolized too rapidly in vivo, despite their effectiveness in cell culture [36,37]. To develop more biologically stable compounds, analogs have been derived from BDCRB, including acetylated, tetrahalogenated benzimidazole D-ribonucleosides, 2-bromo-4,5,6-trichloro-1-(2,3,5-tri-*O*-acetyl-β-d-ribofuranosyl) benzimidazole (BTCRB) and 2,4,5,6-tetrachloro-1-(2,3,5-tri-*O*-acetyl-β-d-ribofuranosyl benzimidazole (Cl4RB), which inhibit DNA cleavage and packaging [38,39]. BTCRB is suspected to inhibit the ATPase activity of pUL56 [38], while Cl4RB is believed to interfere with the interaction between pUL56 and the portal protein pUL104 [40]. These two compounds are shown to be active against VZV, rat cytomegalovirus, and human cytomegalovirus, however Cl4RB has no effect on HHV-1 and the effects of BTCRB are minimal [39].

Raltegravir, an HIV integrase inhibitor, exhibits efficiency against herpes viruses. The effect of raltegravir on herpes viruses is attributed to an inhibition of the large subunit of the viral terminase because it has the same RNase H-like fold as the HIV integrase [31]. Recently however, drug resistance to raltegravir has been traced to HHV-1 UL42 coding for the DNA polymerase accessory factor [41]. Due to the fact that pUL42 is not a part of the terminase, it was determined that raltegravir likely inhibits DNA replication through the polymerase accessory factor rather than DNA cleavage and packaging through the terminase [41]. Letermovir (AIC246 or MK-8228), is another promising new antiviral drug for the treatment of CMV. Letermovir is believed to inhibit the viral terminase complex by targeting pUL56 because L241P, R369S, and C325Y mutations in pUL56 correlate with resistance to letermovir [31,42,43]. The inhibitory effect of letermovir is believed to be distinct due to the lack of cross-resistance of letermovir-resistant CMV strains to benzimidazoles [31,32]. While letermovir has no target-related toxicity, and has a good safety profile, it is specific for human CMV and is ineffective against other viruses, including the remaining herpesviruses [44].

Although numerous antivirals have been developed against HHV, many of these compounds are prone to resistance, have poor bioavailability and high toxicities, and are too specific to be utilized against other HHVs [45,46]. We focus on the most conserved protein in HHVs. We propose DNA packaging terminase pUL15 as a drug target that also could be utilized in other herpesviridae and, possibly, a larger set of viruses utilizing a DNA packaging motor. We also suggest that the hinged region between the two domains of pUL15 is a promising drug target to inhibit its overall function.

## 2. Materials and Methods

### 2.1. Estimated Rate of Change

Eight previously sequenced herpesvirus strains, highlighted in bold in Table 1, were selected from GenBank [47] to be included in genome-wide phylogenetic analyses. These sequences were chosen to represent all major groups of alphaherpesviruses, while also focusing on those found in human, HHV-1 and HHV-2. To conduct phylogenetic analyses, homologous regions of the genome must be identified and aligned. We conservatively interpreted available annotations, excluding any potential homologs in which annotation was unclear, to identify 59 homologs that were then extracted from each genome and compiled into gene-by-gene datasets. Each gene-by-gene dataset was aligned with MAFFT version 7 using the E-INS-i algorithm for the highest level of accuracy [48]. To select the best fitting model of amino acid substitution, we used the Bayesian Information Criterion [49] as implemented in ProtTest version 3.2 [50]. All available 120 amino acid substitution models were tested and, in each case, the Jones92 model with a gamma distribution and invariant sites [51] was preferred. All individual gene datasets were then concatenated and partitioned, based on gene identity, resulting in an alignment with 41,543 amino acids. A Bayesian Markov Chain Monte Carlo (MCMC) analysis with fixed partitioning schemes was performed using MrBayes version 3.2.6 [52] using 4 independent runs of 5 million generations, with 4 Metropolis-coupled chains per run. Fixed partitioning analyses included parameter estimates across all 59 genes. Regarding each partition, all substitution model parameters, with the exceptions of topology and branch lengths, were unlinked across data subsets, allowing each partition to independently estimate relative rates of evolution. Analyses were monitored for convergence by examining trace plots of scalar values in Tracer version 1.6 [53]. Topological convergence was assessed using the average standard deviation of split frequencies technique. To compare the relative rates of evolution across different genes, the inferred rate multipliers from the posterior distribution were plotted using Tracer version 1.6 [53].

### 2.2. Phylogenetic Tree

Twenty-one HHV-1 UL15 gene homologs from various herpes virus species were collected by BLAST [54] with HHV-1 as the query. These sequences, highlighted in italics in Table 1, have E-values <10^−15^ against HHV-1. Amino acid sequences were obtained from the Reference Sequence (RefSeq) database [55]. Multiple sequence alignment was performed with MUSCLE [56]. The ProtTest 2.4 server [57] was used to determine the best fitting substitution model. The LG+I+G+F substitution model scored by AIC was found to be the best suited to the alignment. A phylogenetic tree was generated for the amino acid alignment based on the LG+I+G+F model using maximum likelihood inference preformed on RAxML v8.2.4 [58] with bootstrapping of 100 replicates. Additionally, a Bayesian MCMC estimation was conducted, and a majority-rule consensus tree was generated by MrBayes v3.2.6 [52,59]. The trees were visualized in FigTree v1.4.3 [60] using, GaHV-2 as the outgroup to root the trees. The recovered topologies of the two trees were identical, except for the reverse placement of HHV-4 and BHV-6. Both the bootstrap and posterior probabilities values are displayed on the Bayesian tree [61].

### 2.3. Protein Structure Modeling

The amino acid sequence of HHV-1 pUL15 was obtained from the RefSeq database [55] (GeneID: 2703385). Only a partial crystal structure of the C-terminus of pUL15 (PDB-ID: 4iox) [62] is available in the Protein Data Bank (PDB) [63]. DNA packaging protein gp17 from enterobacteria phage T4 (PDB-ID: 2o0j) [64] was selected by HHPRED [65] as the most reliable template to model the N-terminal domain of pUL15. To properly orient the two proteins, two template structures (2o0j and 4iox) were superposed onto the crystal structure of T4 gp17 (PDB-ID: 3cpe) [66] by Fr-TM-align [67]. Following the structure alignment, a sequence alignment was done using SALIGN [68] to align the sequences of 2o0j and 4iox to the sequence of pUL15. The sequence and structure alignments then were utilized to create the model of pUL15 with MODELLER 9.16 [69], excluding the first 126 amino acids due to the lack of templates to generate a high-quality model of this pUL15 segment.

### 2.4. ATP-Binding Site Prediction

The pUL15 model was screened for potential binding pockets based on evolutionarily related proteins using *e*FindSite [70,71]. The output from *e*FindSite was inserted into *e*SimDock [72], which determined that ATP is most likely to bind to that particular binding site and yielded a complex structure with the ATP docked into the protein. To resolve any possible clashes, the ligand-protein complex was input into LigPlot+ [73]. The free energy required for the ATP binding also was estimated with the distance-scale finite ideal-gas reference (DFIRE) potential [74]. The energy of association between ATP and pUL15 was compared to other proteins bound to ATP selected with PDBePISA (Proteins, Interfaces, Structures and Assemblies) [75].

### 2.5. DNA-Binding Site Prediction

DNA-protein interactions were predicted with DISPLAR [76] and DP-Bind [77] against the sequence of pUL15 as the input. Additionally, a review of the currently available literature discussing potential DNA binding residues in pUL15 was carried out. Subsequently, those residues common between the literature and the sequence-based prediction, as well as residues in the literature that seem to have a particular importance, were identified. A small segment of HHV-1 DNA was created by putting the nucleotide sequence (GenBank ID: JQ673480.1) into 3D-DART, a DNA structure modelling server [78]. This DNA structure, along with the protein model, were input into HADDOCK [16] to generate a three-dimensional model of the DNA-pUL15 complex. Using HADDOCK, pUL15 residues 517, 695, 700, 701, and 620–633 were labeled as active, whereas the first and the last base pairs of the DNA model were selected as non-active.

### 2.6. Cross-Correlation of Residue Fluctuations

To predict residue fluctuations, an elastic network model for pUL15 was built using the Gaussian Network Model (GNM) via the DynOmics online tool [79]. The default cutoff distance of 7.3 Å between GNM nodes was used. The cross-correlation (CC) map shows the extent of a connection between two nodes representing amino acid residues. DynOmics by default includes all the principal modes of structural variations in the CC map. Each mode represents the direction of the concerted motion of the entire system, which is determined upon principal component analysis (PCA) of the covariance matrix.

## 3. Results

### 3.1. Publications on HHV, HIV, and Influenza

**Figure 1:**
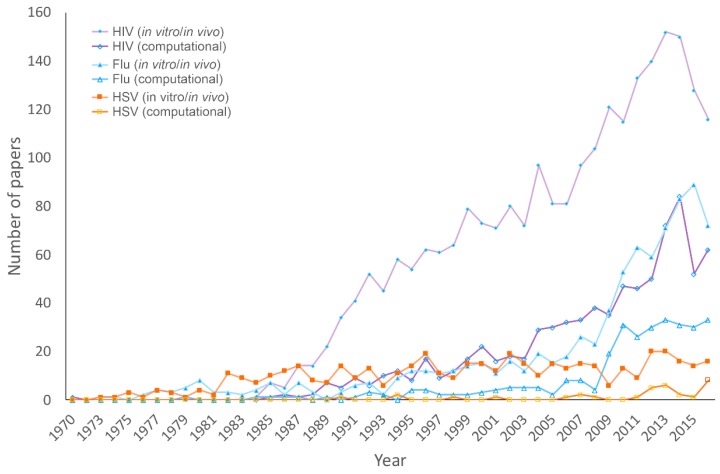
Number of papers concerning viruses published each year since 1970. Papers related to HIV, influenza, and HHV were identified in PubMed using keywords shown in the figure legend (in vitro or in vivo, and computational).

To grasp the amount of research focused on HHV compared to HIV and influenza, we assessed trends in the number of papers indexed by PubMed concerning these viruses. We also looked at how many of these studies utilized computational methods in their research. Figure 1 shows that the number of papers reporting computational research consistently remains fewer than the number of studies employing in vivo or in vitro methods for all three viruses. This is expected, as in vitro and in vivo techniques are traditionally the primary methods for approaching virology research. Even so, the rapid development of technology in genomics and computational science has resulted in more reliable and useful tools that can be used for making relatively accurate predictions to facilitate in vitro and in vivo studies. Essential information for better understanding viruses, including viral structures, interactions, and evolution, which can be used to help develop strategies to combat viruses [80] has been provided by in silico research.

A gap between the number of papers published on HIV compared to HHV and influenza is likely a result of the high mortality rate of HIV and a significant support provided by the World Health Organization Global Special Programme on AIDS [81,82]. The increase in the publication of influenza research papers correlates with flu outbreaks; this is especially true with the outbreak of the highly pathogenic H5n1 in Asia in 2003 and the Swine flu pandemic in 2009. Herpes viruses lack the dramatic peaks in transmission that are seen in influenza and the widespread mortality rate of HIV to spur research, which likely results in comparatively fewer publications. However, the human herpesvirus family includes the pathogens HHV-1 and HHV-2 along with VZV, EBV, CMV, HHV-6, HHV-7, and HHV-8, meaning that the human herpesvirus family is responsible for numerous diseases including roseola, post-transplant infections, neurological disabilities in infants, chicken pox, shingles, lymphoma, nasopharyngeal carcinoma, Hodgkin’s disease, and infectious blisters on the lips and genitals. HHV-1 and HHV-2 alone can result in neonatal infection, keratitis meningitis, encephalitis, and HHV-2 increases the likelihood of HIV infection by three-fold [83]. The ability of HHV to establish latency gives it permanent residence in the body and has made it a difficult target for antiviral strategies [84]. Although there are some antivirals currently available to treat herpesvirus, these therapeutics have been insufficient due to the increasing antiviral resistance.

### 3.2. Relative Substitution Rates for HHV-1 Proteins

**Figure 2:**
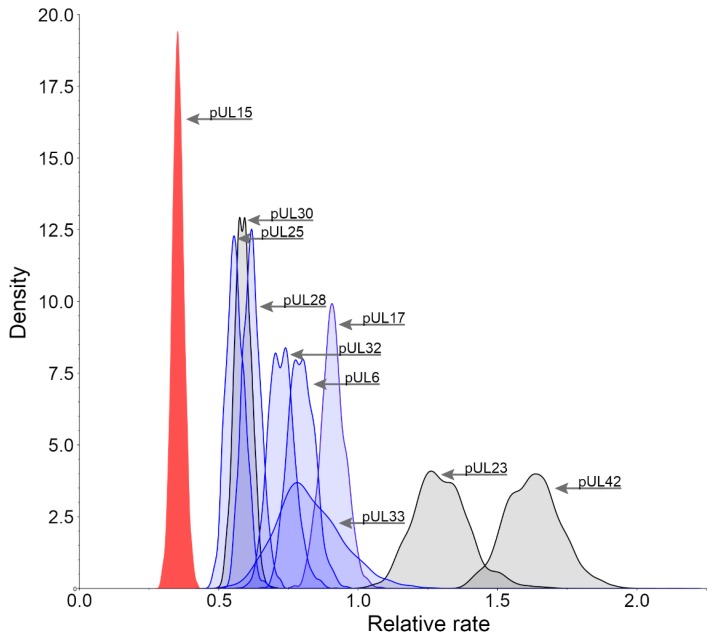
Posterior distributions of relative substitution rates. Protein products of HHV-1 involved in the packaging of DNA (pUL6, pUL15, pUL17, pUL25, pUL28, pUL32, and pUL33) are shown in blue and the protein products of HHV-1 that are currently used as antiviral targets (pUL23, pUL30 and pUL42) are shown in gray. pUL15 (red) has been highlighted to show an extremely conservative rate of change relative to the other packaging related genes.

The analysis of the rate of change of all proteins in the herpesviridae family reveals that those involved in the packaging of DNA are generally the most conserved. These proteins include pUL6, pUL15, pUL17, pUL25, pUL28, pUL32, and pUL33. Mutations or removal of these proteins result in complications in the formation of the virus, typically affecting the ability to successfully cleave and package DNA into its capsid. This makes any of these proteins a potential antiviral target because, once the DNA packaging has been hindered, the spread of new virus particles will be impaired. To determine suitable protein targets, the rates of change were calculated for those protein products of HHV-1 involved in the packaging of DNA. Figure 2 shows that pUL15 (red) is more conserved than not only any other packaging protein in HHV-1 (blue), but also the current drug targets (gray). pUL42 and pUL23, in accord with previous studies [20], are the least conserved out of the proteins which were analyzed, which may explain the resistance that has emerged against raltegravir and acyclovir, respectively. Since pUL15 is one of the most conserved proteins in the entire virus, utilizing it as a model for drug discovery may prove to be highly beneficial. Although letermovir has already paved the way for DNA packaging motor inhibition, it has done so by targeting the pUL28 homolog in CMV and has been observed to only affect CMV. This could be explained by the lesser degree of conservation found in the pUL28 homolog in HHV-1. Targeting a more conserved protein, such as pUL15, may allow for a more inclusive use of antivirals by developing wide spectrum therapies to treat multiple viruses from the HHV family.

### 3.3. Phylogenetic Tree Analysis

**Figure 3:**
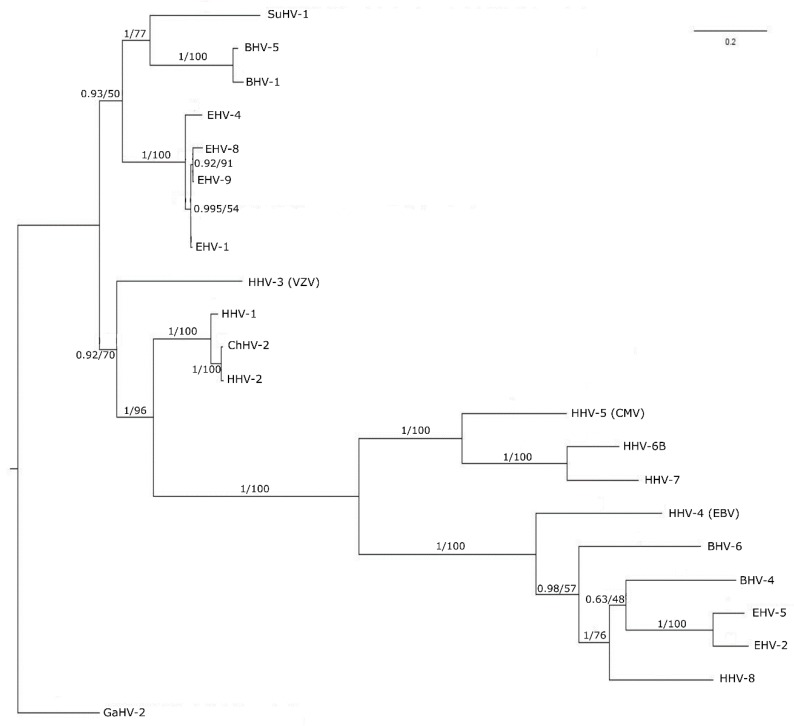
Phylogenetic tree of 21 homologs of HHV-1 pUL15. The tree is rooted on an outgroup GaHV-2. Sequences are labeled by virus abbreviations. Each node shows the posterior probability value from a Bayesian analysis followed by the bootstrap support value reported as percentages from maximum likelihood analysis. The scale bar represents the number of substitutions along each branch.

The highly conserved nature of pUL15 suggests that it should have similarities to its counterparts in other human herpes viruses. The phylogenetic analysis shows the level of similarity amongst HHV-1 UL15 and its human herpes virus homologs within the context of the phylogenetic tree visualized in Figure 3. As expected, HHV-1 UL15 is related most closely to the HHV-2 UL15 homolog. It is noteworthy that HHV-1 and HHV-2 together infect nearly 80% of the world population [85,86]. Our research is focused on HHV-1 as it is the more common of the two viruses affecting 48% of the United States population [87]. HHV-1 and HHV-2, however, are closely related, with an over 80% sequence similarity of their protein-coding regions. HHV-1 also is closely related to the other human herpes viruses, as well as those herpes viruses affecting high-value domesticated animals in the agriculture industry. The close relationship between UL15 homologs of HHV-1 and the other human herpes virus protein sequences is evident by the fact that all of these viruses form a monophyletic clade in the tree. However, the short branch lengths of the tree indicate that, overall, there is a high level of similarity between all of the sequences. The relationship between UL15 in HHV-1 and its homologs suggest that UL15 is indeed a good candidate for creating a structure model, which also can be applied to other closely related proteins.

### 3.4. Structure Model of pUL15

**Figure 4:**
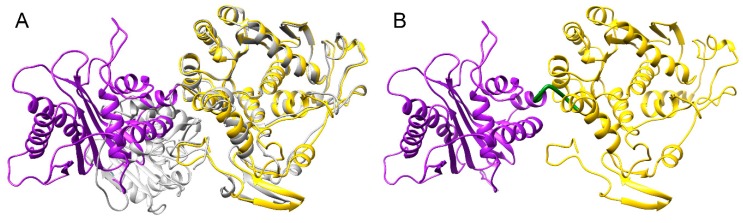
Template-based structure modeling of pUL15. (**A**) Superimposition of pUL15 domains onto the template structure. A superimposition of 4iox (purple) and 2o0j (yellow) onto 3cpe (gray) was used to create the pUL15 model shown in (**B**). A hinge region linking the two domains of pUL15 is colored in green.

To create a full-length model of pUL15, a partial crystal structure of the C-terminal domain of pUL15 was used, whereas the N-terminal domain was modeled based on the DNA packaging protein gp17 from enterobacteria phage T4. Gp17 was chosen due to its high homology to the N-terminal domain of pUL15. To construct an accurate model, the two templates were oriented properly by superimposing the templates onto another structure of T4 gp17 (3cpe), shown as gray ribbons in Figure 4A. The superimposition of 2o0j (yellow ribbons in Figure 4A) onto 3cpe yielded a Cα-RMSD of 1.34 Å and a 100% sequence identity over the aligned region. The superimposition of 4iox (purple ribbons in Figure 4A) onto 3cpe yielded a Cα-RMSD of 3.21 Å and a 10% sequence identity over the aligned region. Similarity scores obtained for 2o0j superposition onto 3cpe indicate a more successful alignment because 2o0j is actually a partial structure of 3cpe. The differences that are evident in the superimposition most likely can be attributed to the conformation that 2o0j takes when it is bound to ATP, as opposed to the conformation in the unbound form. While the scores obtained for 4iox do not show a high similarity to 3cpe, the purpose of this superimposition was simply to orient the templates with respect to each other. The generated model covers residues 127–735 due to the lack of suitable templates to model the first 126 amino acids of pUL15. The model of pUL15 subsequently was subjected to a series of analyses to gain insights into its dynamics and function.

### 3.5. Cross-Correlation between Residue Fluctuations

**Figure 5:**
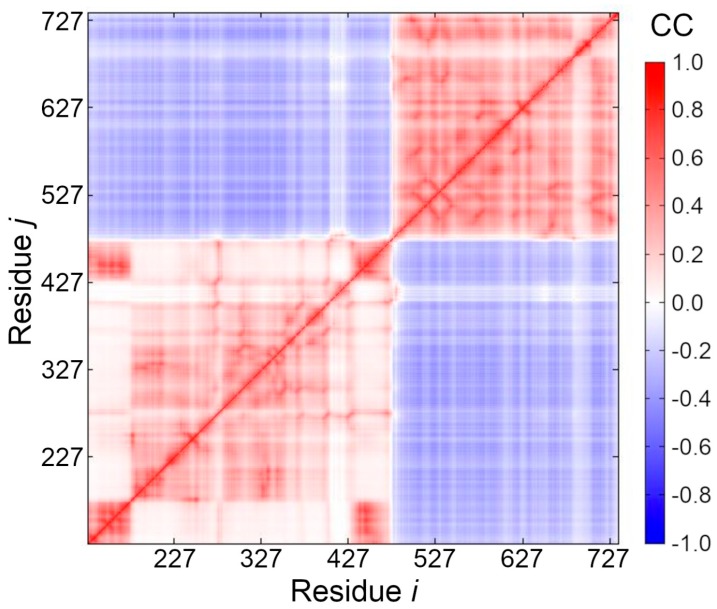
Orientational cross-correlations between residue fluctuations. The cross-correlation (CC) map shows the extent of correlation of the movements between two nodes, ***i*** and ***j***, representing amino acid residues. CC values vary from−1 (fully anticorrelated motions, blue) to +1 (fully correlated motions, red).

To further investigate the distinct domains of pUL15 with respect to their motions, a cross-correlation heat map was generated based on the elastic network model using the DynOmics online server [79]. Figure 5 clearly shows that there are two distinct areas where residue movements are correlated (red color). The first region corresponds to the ATP binding domain (residues 127–466), and the second region corresponds to the DNA binding region (residues 488–735). Moreover, the two domains show anti-correlated movement (blue color), which can generate the force necessary to push or screw the DNA. The heat map also reveals a hinge region that connects the two domains at residues 467–487. We predict that the functions of both domains may rely on the movement of the hinge region (colored in green in Figure 4B), therefore, we expect this hinge region to be a potential allosteric site for drug development.

### 3.6. Ligand-Binding Site in pUL15

**Figure 6:**
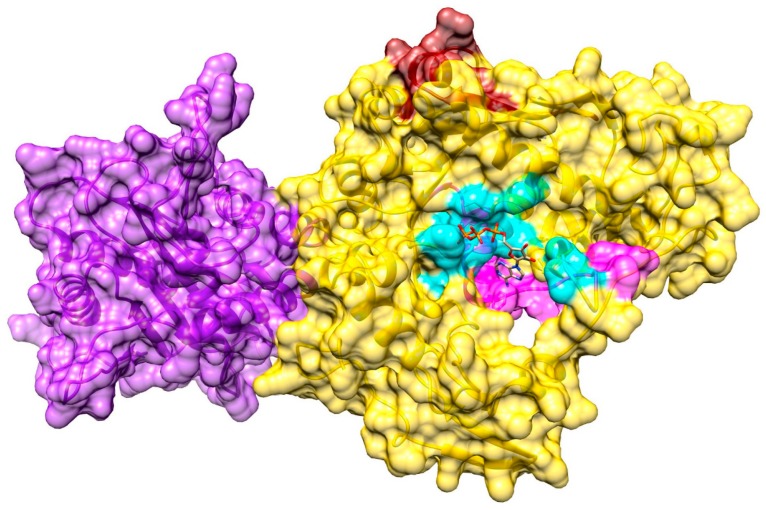
Model of pUL15 complexed with ATP. The N-terminal of the protein is yellow, and the C-terminal is purple. The predicted ATP-binding site is shown in cyan, whereas the two Walker boxes are colored in magenta (Walker box A) and brick red (Walker box B).

A putative binding site in the pUL15 model was predicted by *e*FindSite with a 97.9% confidence. This site is comprised of residues A244, A245, V246, T254, V255, L257, R260, F279, R280, G281, I282, K283, I284, G285, and G319, which are highlighted in cyan in Figure 6. All these residues lie on the N-terminal domain of the protein. Additionally, both of the template proteins were bound to ligands, 2o0j was bound to ADP while 2o0h was bound to ATP. Furthermore, fingerprint-based virtual screening conducted with *e*FindSite suggests that this binding pocket is likely an ATP-binding site. Interestingly, as shown in Figure 6, the predicted binding site also was found next to two ATP binding motifs known as Walker A (magenta) and Walker B (brick red). The Walker A motif has been suggested to play a critical role in the hydrolysis and binding of ATP. It works in conjunction with the Walker B motif which is responsible for coordinating the magnesium ion of the Mg^2+^—ATP complex. These two motifs work together to properly orient the phosphates of ATP for hydrolysis. The binding pocket for ATP have been found upstream from the Walker A motif of gp17 of the T4 bacteriophage, which was used as a template for the protein model. Walker A and Walker B boxes are found at residues 249−272 and 345−362, respectively [88]. Another study suggested somewhat narrower ranges for Walker A (residues 258–265) and Walker B (residues 352–357) motifs [89]. The predicted ATP binding site is consistent with several residues found upstream of, and overlapping with, the Walker A box, further indicating that the binding site was predicted properly in the pUL15 model.

### 3.7. Model of pUL15 Complexed with ATP

**Figure 7:**
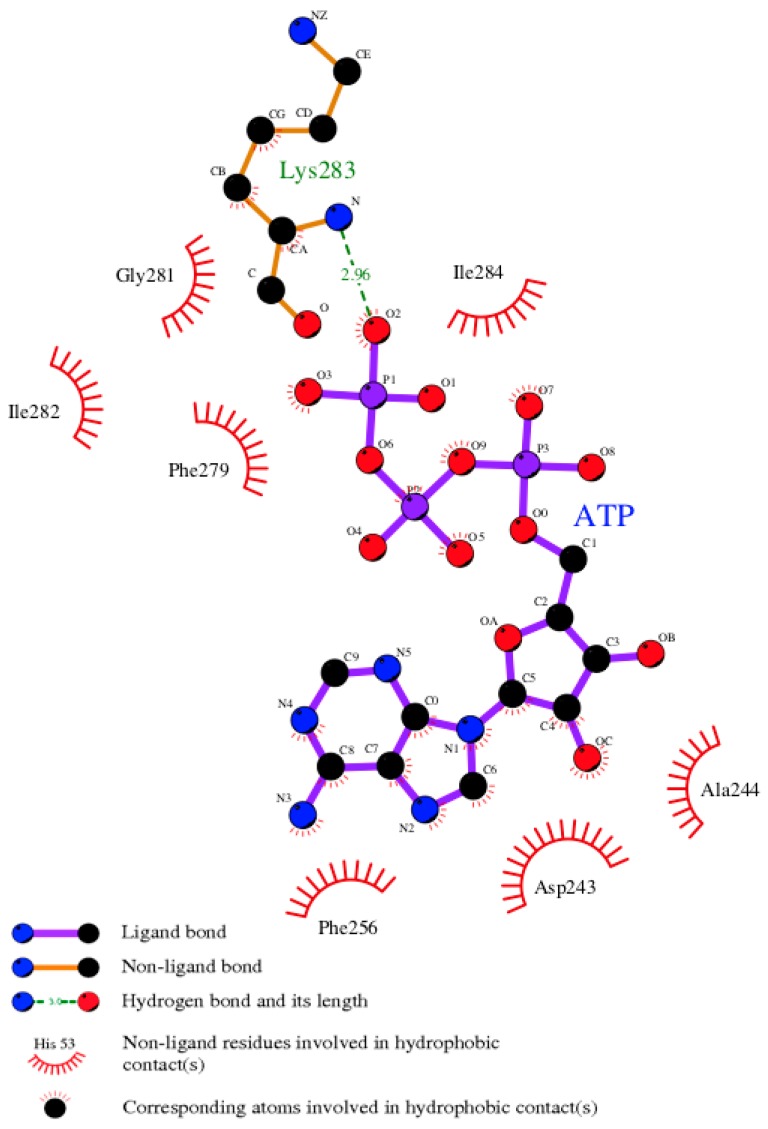
Ligand-protein contacts in the pUL15-ATP complex. Residues in the modeled pUL15 binding pocket interacting with the docked ATP molecule are shown. The various types of bonds are indicated in the figure legend.

**Figure 8:**
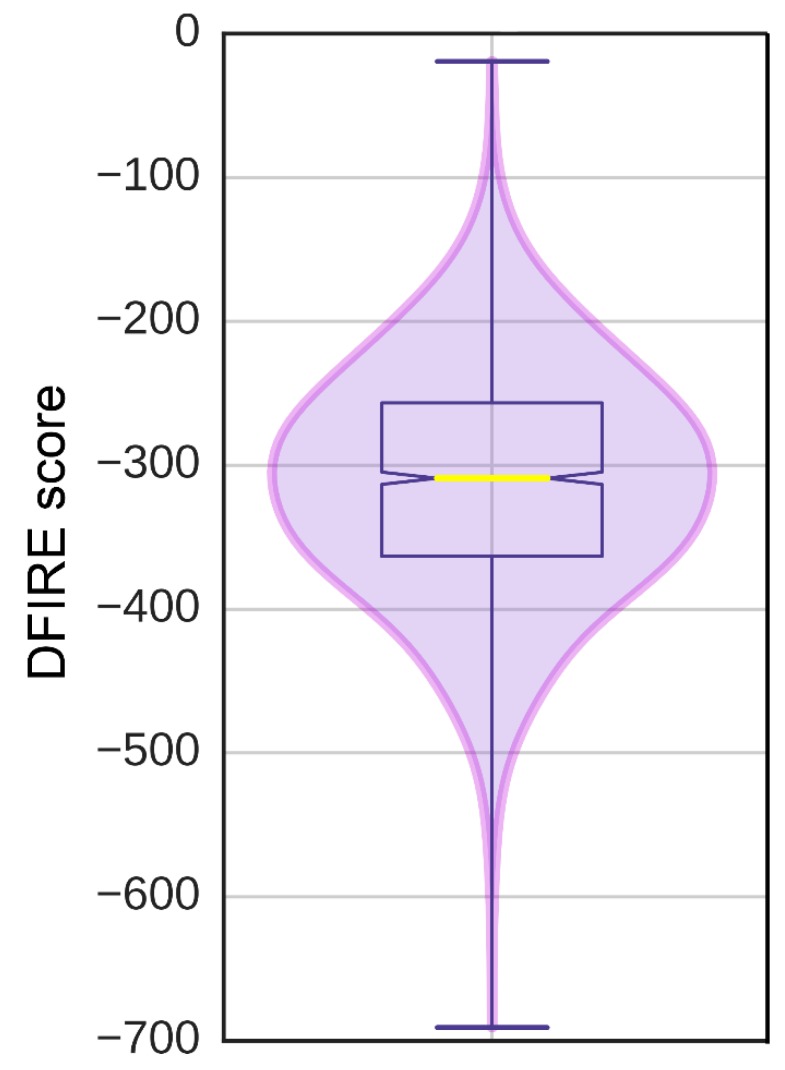
Distribution of DFIRE scores for protein-ATP complexes. Scores were calculated for the experimental structures of 1552 protein-ATP complexes.

The *e*FindSite prediction strongly indicates that the putative binding site accommodates ATP. To further investigate this finding, a pUL15-ATP complex was modeled with *e*SimDock. Encouragingly, *e*SimDock reported a fitness score of 0.51, a binding probability of 0.84, and an estimated p*K*_d_ of 6.11. These scores corroborate the *e*FindSite prediction that ATP is the likely ligand of the predicted binding site. The ATP molecule that was docked contained a few minor clashes between the ligand and the protein, which may account for the somewhat low fitness score. To remove the clashes, binding residues within 4.5 Å from any ATP atom were remodeled in the presence of ATP. The analysis of the ATP binding pose in the refined pUL15-ATP complex reveals a hydrogen bond between a phosphate moiety and K283, and numerous hydrophobic contacts with residues D243, A244, F256, F279, G281, I282, and I284 (Figure 7). Although, ADP in the crystal structure of g17 forms more extensive hydrogen-bonding interactions, the Szymkiewicz−Simpson coefficient [90] between the binding pockets of g17 and pUL15 is only 0.27 indicating a limited binding residue conservation. To determine how the binding energy of the modeled pUL15-ATP complex compares to other protein-ATP complexes, DFIRE scores were calculated for the modeled complex and a dataset of 1553 other protein-ATP complexes. Figure 8 shows that the mean DFIRE score for experimental protein-ATP complexes is −311.35, whereas values at the first, second, and third quartiles are −363.31, −309.00, and −256.62, respectively. The DFIRE score for the modeled pUL15-ATP complex is −255.13, which corresponds to the upper range of the third quartile of the distribution of DFIRE scores across protein-ATP complexes. This shows that the interaction energy between the ATP molecule and pUL15 is comparable to that in many experimental complex structures involving ATP.

### 3.8. Model of pUL15 Bound to DNA

**Figure 9:**
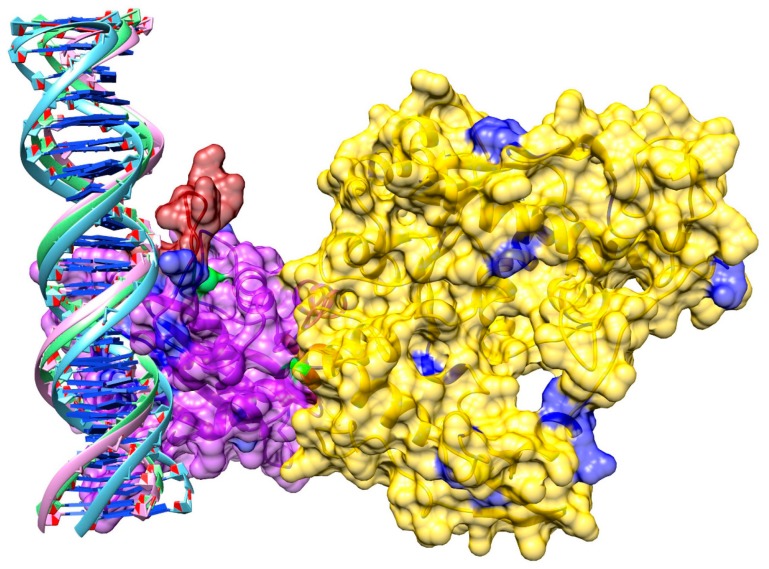
Model of pUL15 complexed with dsDNA. The N-terminal of the protein is yellow, and the C-terminal is purple. DNA binding residues predicted with DISPLAR (blue), DP-Bind (red) and both programs (green) are shown.

A sequence-based analysis of DNA binding was conducted with DP-Bind and DISPLAR. Since sequence-based predictions may not always be reliable, a review of currently available literature discussing potential DNA binding residues in pUL15 was conducted. To test these predictions, we docked DNA to the pUL15 model. We computationally created a double-stranded (ds)DNA with a 3D-DART using an HHV-1 sequence and docked it to the protein with HADDOCK. The third cluster of the top five results was selected as it was the most consistent with binding residues reported by DP-Bind, DISPLAR, and the published literature. A detailed model of pUL15 bound to DNA shown in Figure 9 reveals three loop structures flanking the DNA. Two are flanking the DNA on one side, while the third is positioned on the other side of the DNA. Added to the heat map described above, the DynOmics online server also generated a molecular motion using an Anisotropic Network Model (ANM), which provides information on how protein residues are predicted to move. A movie showing the molecular motion of pUL15 predicted based on its cross-correlation map is included as Additional file 1 (Supplementary Material). These results indicate that the protein is moving in a manner perpendicular to the DNA, supporting a hypothesis that the DNA movement likely involves either rotation or revolution, rather than a simple linear motion.

## 4. Discussion

Many dsDNA viruses utilize a motor to package their DNA into an empty capsid and, therefore, may be classified under type II DNA-packaging. However, the exact mechanism of the DNA packaging with detailed movements of the machinery are unknown. Most dsDNA viruses contain packaging proteins that are composed of two separate domains, an ATPase domain as well as a DNA-binding/nuclease domain. These two functions are linked together, which is necessary for proper DNA packaging to occur. Several hypotheses were formulated to try to answer this question. One popular theory involves the idea that proteins in the packaging motor push the DNA into the capsid in a linear manner. Considering the linear hypothesis, it is believed that the protein utilizes the hydrolysis of ATP to clamp onto the DNA and push it up into the empty capsid [91]. This mechanism begins with the DNA-binding domain binding the viral DNA. Once the DNA is bound, the DNA-binding domain moves the viral DNA closer to the ATPase domain of the same protein. A conformational change takes place in the ATPase domain, prepping the ATPase active site for hydrolysis. The subsequent hydrolysis of ATP causes another conformational change that moves the DNA-binding domain in such a way that it pushes the viral DNA into the capsid. When ADP and Pi are released, the DNA-binding domain releases the DNA and returns to its original conformation. When the DNA is released from the DNA-binding domain, it ends up being bound to the DNA-binding domain of an adjacent subunit [92].

A second hypothesis, the rotation theory, states that the DNA is packaged into the capsid with a screw-like motion as the packaging proteins rotate to facilitate this motion. More specifically, it refers to the possibility of a connecter protein in the packaging motor rotating around the viral DNA and, therefore, using this movement to screw the DNA into the capsid. However, more recent studies suggest that dsDNA viruses are unlikely to utilize the rotational mechanism. It was reported that rotational motors have relatively small channels that are typically smaller than the width of dsDNA, so when the DNA goes through that channel, it would require the dsDNA to split into ssDNA [91]. A more plausible hypothesis has been suggested where the packaging motors utilize a revolving mechanism instead. This means that rather than the proteins rotating to push the DNA through, the proteins are relatively stationary and the DNA revolves around the channel due to the backbone interactions with the walls of the channel. Revolution motors have been found to have diameters larger than the width of dsDNA, allowing the DNA to go through the revolving mechanism [91].

Finally, the “Scrunchworm Hypothesis” considers DNA to be what provides the energy for its own translocation rather than the protein doing all of the work [93]. During the process of packaging, proteins bind to the DNA and remove it from the solvent, causing its dehydration and compression. The energy stored in the DNA at this compressed state then is responsible for the ultimate movement of the DNA into the empty capsid.

The GNM analysis of pUL15 indicates that it contains two domains connected by a hinge that move in opposite directions of one another. When dsDNA was docked onto our model, we found that the DNA was surrounded by three loop structures flanking it on both sides. Further, the ANM model suggests that the movement of the protein upon ATP hydrolysis most likely would be perpendicular to that of the DNA, contrary to the linear theory. Our data indicates that the packaging of DNA would follow more closely with either the rotation or revolution theory. Due to the limitations of the rotation theory, particularly the small size of the channel for the dsDNA to pass through, it is more likely that the DNA is packaged according to the revolution theory in HHV-1 due to its abundant space for the dsDNA to pass through the channel.

Proteins directly involved in the propagation of the concatemeric viral DNA into the capsid have a significant potential as drug targets due to their essential role in the formation of new virus particles. Out of all of the packaging proteins, terminase proteins maintain the crucial functions of utilizing ATP hydrolysis to push the DNA into the capsid and cleave it once packaging is complete. This makes them promising drug targets because there are multiple functions that may be disturbed to prevent the completion of virus particles. Using this information, the hinge region of pUL15, connecting the DNA and ATP binding domains, is an attractive allosteric target. While targeting DNA- and ATP- binding sites may inhibit the function of this protein, these binding pockets are not unique to the virus. Therefore, we hypothesize that the hinge region can provide an allosteric target to alter the conformational change in pUL15, preventing the movement of the dsDNA into the capsid. It is a favorable site for drug design as multiple HHVs share the same feature in their packaging motor, yet it is specific to the virus and absent in host proteins.

## 5. Conclusions

Through evolutionary analysis, we determined that focusing on HHV-1 would be the most effective for targeted drug development, not just because it is the most abundant form of HHV, but also because it is closely related to the other HHVs. Our findings suggest that the most ideal target would be the packaging motor due to its crucial role in the lifecycle of the virus and a high conservation throughout all herpesviridae. The rate of change analysis revealed that the large terminase pUL15 is the most conserved, not just in the packaging motor, but in all of the viral proteins. Since only a partial experimental structure of this protein is available currently, we constructed a full-length model to analyze its molecular functions. Using this model, we were able to confidently identify putative ATP and DNA binding sites. Additionally, we used the Gaussian network model to predict the correlation between residue fluctuations, which not only supported the presence of two distinct domains in the protein, but also revealed a hinge region between the ATP- and DNA-binding domains. Further, the anisotropic network model was used to observe the probable movements of the protein and indicated that the HHV-1 packaging motor likely utilizes a revolutionary mechanism to package DNA., It will be a promising target for antiviral drugs against HHVs if the predicted hinge region can be confirmed in subsequent studies.

## Figures and Tables

**Table 1:** Herpesvirus pUL15 orthologs analyzed in this study. Species in bold were used to estimate the rate of change, whereas those in italics were used to construct the phylogenetic tree.

Herpesvirus	Abbreviation	Accession Number	% Identity with HHV-1
***Human Alphaherpesvirus 1***	HHV-1	YP 009137089.1	100.0%
***Human Alphaherpesvirus 2***	HHV-2	YP_009137166.1	94.8%
*Human Alphaherpesvirus 3*	HHV-3	NP_040165.1	59.0%
*Human Gammaherpesvirus 4*	HHV-4	YP 401690.1	32.2%
*Human Betaherpesvirus Type 5*	HHV-5	YP_081537.1	36.0%
*Human Betaherpesvirus 6B*	HHV-6B	NP_050241.1	34.5%
*Human Betaherpesvirus 7*	HHV-7	YP_073802.1	33.0%
*Human Gammaherpesvirus 8*	HHV-8	YP_001129382.1	31.6%
***Chimpanzee Herpesvirus Strain 105640***	ChHV-2	YP_009011001.1	95.0%
**Cercopithecine Herpesvirus 2**	CeHV-2	YP_164457.1	87.3%
*Equid Alphaherpesvirus 1*	EHV-1	YP_053090.1	63.9%
*Equid Gammaherpesvirus 2*	EHV-2	NP_042630.2	30.6%
***Equid Alphaherpesvirus 4***	EHV-4	NP_045262.1	63.7%
*Equid Gammaherpesvirus 5*	EHV-5	YP_009118419.1	31.6%
*Equid Alphaherpesvirus 8*	EHV-8	YP_006273027.1	29.6%
*Equid Alphaherpesvirus 9*	EHV-9	YP_002333526.2	64.1%
**Felid Herpesvirus 1**	FHV-1	YP_003331564.1	64.3%
**Fruit bat Alphaherpesvirus 1**	FBAHV-1	YP_009042077.1	82.6%
***Bovine Alphaherpesvirus 1***	BHV-1	NP 045342.1	60.4%
*Bovine Gammaherpesvirus 4*	BHV-4	NP 076521.1	31.3%
*Bovine Alphaherpesvirus 5*	BHV-5	YP 003662508.1	60.6%
*Bovine Gammaherpesvirus 6*	BHV-6	YP 009042007.1	31.0%
*Gallid Alphaherpesvirus 2*	GaHV-2	AAF66813.1	53.9%
*Suid Alphaherpesvirus 1*	SuHV-1	YP 068358.1	55.2%

## Supplementary Materials

Supplementary materials are available on line at https://www.mdpi.com/2218-273X/9/10/603/s1.

## References

[1] Wald A., Corey L., Arvin A., Campadelli-Fiume G., Mocarski E. (2007). Persistence in the population: Epidemiology, transmission. Human Herpesviruses: Biology, Therapy, and Immunoprophylaxis.

[2] Fishman J.A., Emery V., Freeman R., Pascual M., Rostaing L., Schlitt H.J., Sgarabotto D., Torre-Cisneros J., Uknis M.E. (2007). Cytomegalovirus in transplantation - challenging the status quo. Clin. Transplant.

[3] Penkert R.R., Kalejta R.F. (2011). Tegument protein control of latent herpesvirus establishment and animation. Herpesviridae.

[4] Chisholm C., Lopez L. (2011). Cutaneous infections caused by herpesviridae: A review. Arch. Pathol. Lab. Med..

[5] Grinde B. (2013). Herpesviruses: Latency and reactivation - viral strategies and host response. J. Oral. Microbiol..

[6] Kotton C.N. (2010). Management of cytomegalovirus infection in solid organ transplantation. Nat. Rev. Nephrol..

[7] Whitley R.J., Baron S. (1996). Herpesviruses. Medical Microbiology.

[8] Boppana S.B., Fowler K.B., Britt W.J., Stagno S., Pass R.F. (1999). Symptomatic congenital cytomegalovirus infection in infants born to mothers with preexisting immunity to cytomegalovirus. Pediatrics.

[9] Boppana S.B., Pass R.F., Britt W.J., Stagno S., Alford C.A. (1992). Symptomatic congenital cytomegalovirus infection: Neonatal morbidity and mortality. Pediatr. Infect. Dis. J..

[10] Coll O., Benoist G., Ville Y., Weisman L.E., Botet F., Anceschi M.M., Greenough A., Gibbs R.S., Carbonell-Estrany X., Group W.P.I.W. (2009). Guidelines on CMV congenital infection. J. Perinat. Med..

[11] Peritz D.C., Duncan C., Kurek K., Perez-Atayde A.R., Lehmann L.E. (2008). Visceral varicella zoster virus (VZV) after allogeneic hematopoietic stem cell transplant (HSCT) in pediatric patients with chronic graft-versus-host disease (cGVHD). J. Pediatr. Hematol. Oncol..

[12] Bonnet M., Guinebretiere J.M., Kremmer E., Grunewald V., Benhamou E., Contesso G., Joab I. (1999). Detection of Epstein-Barr virus in invasive breast cancers. J. Natl. Cancer Inst..

[13] Wong M., Pagano J.S., Schiller J.T., Tevethia S.S., Raab-Traub N., Gruber J. (2002). New associations of human papillomavirus, Simian virus 40, and Epstein-Barr virus with human cancer. J. Natl. Cancer Inst..

[14] Schulz T.F. (2006). The pleiotropic effects of Kaposi’s sarcoma herpesvirus. J. Pathol..

[15] Sunil M., Reid E., Lechowicz M.J. (2010). Update on HHV-8-Associated Malignancies. Curr. Infect. Dis. Rep..

[16] McGeoch D.J., Dalrymple M.A., Davison A.J., Dolan A., Frame M.C., McNab D., Perry L.J., Scott J.E., Taylor P. (1988). The complete DNA sequence of the long unique region in the genome of herpes simplex virus type 1. J. Gen. Virol..

[17] Macdonald S.J., Mostafa H.H., Morrison L.A., Davido D.J. (2012). Genome sequence of herpes simplex virus 1 strain KOS. J. Virol..

[18] Macdonald S.J., Mostafa H.H., Morrison L.A., Davido D.J. (2012). Genome sequence of herpes simplex virus 1 strain McKrae. J. Virol..

[19] Davison A.J., Campadelli-Fiume A.A., Mocarski G.E. (2007). Comparative analysis of the genomes. Human Herpesviruses: Biology, Therapy, and Immunoprophylaxis.

[20] Karamitros T., Harrison I., Piorkowska R., Katzourakis A., Magiorkinis G., Mbisa J.L. (2016). De novo assembly of human herpes virus type 1 (HHV-1) genome, mining of non-canonical structures and detection of novel drug-resistance mutations using short- and long-read next generation sequencing technologies. PLoS ONE.

[21] Andrei G., Snoeck R. (2013). Herpes simplex virus drug-resistance: New mutations and insights. Curr. Opin. Infect. Dis..

[22] Razonable R.R. (2011). Antiviral drugs for viruses other than human immunodeficiency virus. Mayo. Clin. Proc..

[23] Griffiths P.D., Boeck M., Arvin A., Campadelli-Fiume G., Mocarski E., Moore P.S., Roizman B., Whitley R., Yamanishi K. (2007). Antiviral therapy for human cytomegalovirus. Human Herpesviruses: Biology, Therapy, and Immunoprophylaxis.

[24] Kimberlin D.W., Whitley R.J., Arvin A., Campadelli-Fiume G., Mocarski E., Moore P.S., Roizman B., Whitley R., Yamanishi K. (2007). Antiviral therapy of HSV-1 and -2. Human Herpesviruses: Biology, Therapy, and Immunoprophylaxis.

[25] Turner N., Strand A., Grewal D.S., Cox G., Arif S., Baker A.W., Maziarz E.K., Saullo J.H., Wolfe C.R. (2019). Use of letermovir as salvage therapy for drug-resistant cytomegalovirus retinitis. Antimicrob. Agents Chemother..

[26] Morfin F., Thouvenot D. (2003). Herpes simplex virus resistance to antiviral drugs. J. Clin. Virol..

[27] Lurain N.S., Chou S. (2010). Antiviral drug resistance of human cytomegalovirus. Clin. Microbiol. Rev..

[28] Schreiber A., Harter G., Schubert A., Bunjes D., Mertens T., Michel D. (2009). Antiviral treatment of cytomegalovirus infection and resistant strains. Expert. Opin. Pharmacother..

[29] Yang L., Yang Q., Wang M., Jia R., Chen S., Zhu D., Liu M., Wu Y., Zhao X., Zhang S. (2019). Terminase large subunit provides a new drug target for herpesvirus treatment. Viruses.

[30] Dittmer A., Woskobojnik I., Adfeldt R., Drach J.C., Townsend L.B., Voigt S., Bogner E. (2017). Tetrahalogenated benzimidazole D-ribonucleosides are active against rat cytomegalovirus. Antiviral. Res..

[31] Goldner T., Hewlett G., Ettischer N., Ruebsamen-Schaeff H., Zimmermann H., Lischka P. (2011). The novel anticytomegalovirus compound AIC246 (Letermovir) inhibits human cytomegalovirus replication through a specific antiviral mechanism that involves the viral terminase. J. Virol..

[32] Melendez D.P., Razonable R.R. (2015). Letermovir and inhibitors of the terminase complex: A promising new class of investigational antiviral drugs against human cytomegalovirus. Infect. Drug Resist..

[33] Yang K., Dang X., Baines J.D. (2017). A domain of herpes simplex virus pUL33 required to release monomeric viral genomes from cleaved concatemeric DNA. J. Virol.

[34] Krosky P.M., Underwood M.R., Turk S.R., Feng K.W., Jain R.K., Ptak R.G., Westerman A.C., Biron K.K., Townsend L.B., Drach J.C. (1998). Resistance of human cytomegalovirus to benzimidazole ribonucleosides maps to two open reading frames: UL89 and UL56. J. Virol..

[35] Underwood M.R., Harvey R.J., Stanat S.C., Hemphill M.L., Miller T., Drach J.C., Townsend L.B., Biron K.K. (1998). Inhibition of human cytomegalovirus DNA maturation by a benzimidazole ribonucleoside is mediated through the UL89 gene product. J. Virol..

[36] Biron K.K. (2006). Antiviral drugs for cytomegalovirus diseases. Antiviral. Res..

[37] Good S.S., Owens B.S., Townsend L.B., Drach J.C. (1994). The disposition in rats and monkey of 2-bromo-5,6-dichloro-1-(ß-D-ribofuranosyl)-benzimidazole (BDCRB) and its 2,5,6-trichloro conger (TCRB). Antiviral. Res..

[38] Hwang J.S., Kregler O., Schilf R., Bannert N., Drach J.C., Townsend L.B., Bogner E. (2007). Identification of acetylated, tetrahalogenated benzimidazole D-ribonucleosides with enhanced activity against human cytomegalovirus. J. Virol..

[39] Hwang J.S., Schilf R., Drach J.C., Townsend L.B., Bogner E. (2009). Susceptibilities of human cytomegalovirus clinical isolates and other herpesviruses to new acetylated, tetrahalogenated benzimidazole D-ribonucleosides. Antimicrob. Agents Chemother..

[40] Dittmer A., Drach J.C., Townsend L.B., Fischer A., Bogner E. (2005). Interaction of the putative human cytomegalovirus portal protein pUL104 with the large terminase subunit pUL56 and its inhibition by benzimidazole-D-ribonucleosides. J. Virol..

[41] Zhou B., Yang K., Wills E., Tang L., Baines J.D. (2014). A mutation in the DNA polymerase accessory factor of herpes simplex virus 1 restores viral DNA replication in the presence of raltegravir. J. Virol..

[42] Goldner T., Zimmermann H., Lischka P. (2015). Phenotypic characterization of two naturally occurring human Cytomegalovirus sequence polymorphisms located in a distinct region of ORF UL56 known to be involved in in vitro resistance to letermovir. Antiviral. Res..

[43] Cherrier L., Nasar A., Goodlet K.J., Nailor M.D., Tokman S., Chou S. (2018). Emergence of letermovir resistance in a lung transplant recipient with ganciclovir-resistant cytomegalovirus infection. Am. J. Transplant..

[44] Marschall M., Stamminger T., Urban A., Wildum S., Ruebsamen-Schaeff H., Zimmermann H., Lischka P. (2012). In vitro evaluation of the activities of the novel anticytomegalovirus compound AIC246 (letermovir) against herpesviruses and other human pathogenic viruses. Antimicrob. Agents Chemother..

[45] Caruso Brown A.E., Cohen M.N., Tong S., Braverman R.S., Rooney J.F., Giller R., Levin M.J. (2015). Pharmacokinetics and safety of intravenous cidofovir for life-threatening viral infections in pediatric hematopoietic stem cell transplant recipients. Antimicrob. Agents Chemother..

[46] Chen K., Cheng M.P., Hammond S.P., Einsele H., Marty F.M. (2018). Antiviral prophylaxis for cytomegalovirus infection in allogeneic hematopoietic cell transplantation. Blood Adv..

[47] Clark K., Karsch-Mizrachi I., Lipman D.J., Ostell J., Sayers E.W. (2016). GenBank. Nucleic Acids Res..

[48] Katoh K., Standley D.M. (2013). MAFFT multiple sequence alignment software version 7: Improvements in performance and usability. Mol. Biol. Evol..

[49] Schwarz G. (1978). Estimating the dimension of a model. Ann. Stat..

[50] Darriba D., Taboada G.L., Doallo R., Posada D. (2011). ProtTest 3: Fast selection of best-fit models of protein evolution. Bioinformatics.

[51] Jones D.T., Taylor W.R., Thornton J.M. (1992). The rapid generation of mutation data matrices from protein sequences. Comput. Appl. Biosci..

[52] Ronquist F., Huelsenbeck J.P. (2003). MrBayes 3: Bayesian phylogenetic inference under mixed models. Bioinformatics.

[53] Rambaut A., Drummond A.J., Xie D., Baele G., Suchard M.A. (2018). Posterior summarization in bayesian phylogenetics using Tracer 1.7. Syst. Biol..

[54] Altschul S.F., Gish W., Miller W., Myers E.W., Lipman D.J. (1990). Basic local alignment search tool. J. Mol. Biol..

[55] O’Leary N.A., Wright M.W., Brister J.R., Ciufo S., Haddad D., McVeigh R., Rajput B., Robbertse B., Smith-White B., Ako-Adjei D. (2016). Reference sequence (RefSeq) database at NCBI: Current status, taxonomic expansion, and functional annotation. Nucleic Acids Res..

[56] Edgar R.C. (2004). MUSCLE: Multiple sequence alignment with high accuracy and high throughput. Nucleic Acids Res..

[57] Abascal F., Zardoya R., Posada D. (2005). ProtTest: Selection of best-fit models of protein evolution. Bioinformatics.

[58] Stamatakis A. (2014). RAxML version 8: A tool for phylogenetic analysis and post-analysis of large phylogenies. Bioinformatics.

[59] Huelsenbeck J.P., Ronquist F., Nielsen R., Bollback J.P. (2001). Bayesian inference of phylogeny and its impact on evolutionary biology. Science.

[60] Fig Tree, Molecular Evolution, Phylogenetics and Epidemiology. http://tree.bio.ed.ac.uk/software/figtree.

[61] Inkscape, Draw Freely. https://inkscape.org/..

[62] Selvarajan Sigamani S., Zhao H., Kamau Y.N., Baines J.D., Tang L. (2013). The structure of the herpes simplex virus DNA-packaging terminase pUL15 nuclease domain suggests an evolutionary lineage among eukaryotic and prokaryotic viruses. J. Virol..

[63] Berman H.M., Battistuz T., Bhat T.N., Bluhm W.F., Bourne P.E., Burkhardt K., Feng Z., Gilliland G.L., Iype L., Jain S. (2002). The Protein Data Bank. Acta Crystallogr. D Biol. Crystallogr..

[64] Sun S., Kondabagil K., Gentz P.M., Rossmann M.G., Rao V.B. (2007). The structure of the ATPase that powers DNA packaging into bacteriophage T4 procapsids. Mol. Cell..

[65] Zimmermann L., Stephens A., Nam S.Z., Rau D., Kubler J., Lozajic M., Gabler F., Soding J., Lupas A.N., Alva V. (2018). A completely reimplemented MPI Bioinformatics Toolkit with a new HHpred server at its core. J. Mol. Biol..

[66] Sun S., Kondabagil K., Draper B., Alam T.I., Bowman V.D., Zhang Z., Hegde S., Fokine A., Rossmann M.G., Rao V.B. (2008). The structure of the phage T4 DNA packaging motor suggests a mechanism dependent on electrostatic forces. Cell.

[67] Pandit S.B., Skolnick J. (2008). Fr-TM-align: A new protein structural alignment method based on fragment alignments and the TM-score. BMC Bioinformatics.

[68] Braberg H., Webb B.M., Tjioe E., Pieper U., Sali A., Madhusudhan M.S. (2012). SALIGN: A web server for alignment of multiple protein sequences and structures. Bioinformatics.

[69] Webb B., Sali A. (2014). Comparative protein structure modeling using MODELLER. Curr. Protoc. Protein Sci..

[70] Brylinski M., Feinstein W.P. (2013). eFindSite: Improved prediction of ligand binding sites in protein models using meta-threading, machine learning and auxiliary ligands. J. Comput. Aided. Mol. Des..

[71] Feinstein W.P., Brylinski M. (2014). eFindSite: Enhanced fingerprint-based virtual screening against predicted ligand binding sites in protein models. Mol. Inform..

[72] Brylinski M. (2013). Nonlinear scoring functions for similarity-based ligand docking and binding affinity prediction. J. Chem. Inf. Model..

[73] Laskowski R.A., Swindells M.B. (2011). LigPlot+: Multiple ligand-protein interaction diagrams for drug discovery. J. Chem. Inf. Model..

[74] Zhang C., Liu S., Zhu Q., Zhou Y. (2005). A knowledge-based energy function for protein-ligand, protein-protein, and protein-DNA complexes. J. Med. Chem..

[75] Krissinel E., Henrick K. (2007). Inference of macromolecular assemblies from crystalline state. J. Mol. Biol..

[76] Tjong H., Zhou H.X. (2007). DISPLAR: An accurate method for predicting DNA-binding sites on protein surfaces. Nucleic Acids Res..

[77] Hwang S., Gou Z., Kuznetsov I.B. (2007). DP-Bind: A web server for sequence-based prediction of DNA-binding residues in DNA-binding proteins. Bioinformatics.

[78] Van Dijk M., Bonvin A.M. (2009). 3D-DART: A DNA structure modelling server. Nucleic Acids Res..

[79] Li H., Chang Y.Y., Lee J.Y., Bahar I., Yang L.W. (2017). DynOmics: Dynamics of structural proteome and beyond. Nucleic Acids Res..

[80] Sato H., Yokoyama M., Toh H. (2013). Genomics and computational science for virus research. Front Microbiol..

[81] Mann J.M. (1987). The World Health Organization’s global strategy for the prevention and control of AIDS. West J. Med..

[82] World Health Organization (2016). Global Health Sector Strategy on HIV, 2016–2021.

[83] Johnston C., Gottlieb S.L., Wald A. (2016). Status of vaccine research and development of vaccines for herpes simplex virus. Vaccine.

[84] Deshpande S.P., Kumaraguru U., Rouse B.T. (2000). Why do we lack an effective vaccine against herpes simplex virus infections?. Microbes. Infect..

[85] Looker K.J., Magaret A.S., Turner K.M., Vickerman P., Gottlieb S.L., Newman L.M. (2015). Global estimates of prevalent and incident herpes simplex virus type 2 infections in 2012. PLoS ONE.

[86] World Health Organization. https://www.who.int/news-room/fact-sheets/detail/herpes-simplex-virus.

[87] McQuillan G., Kruszon-Moran D., Flagg E.W., Paulose-Ram R. (2018). In Prevalence of Herpes Simplex Virus Type 1 and Type 2 in Persons Aged 14-49: USA, 2015-2016. NCHS Data Brief..

[88] Przech A.J., Yu D., Weller S.K. (2003). Point mutations in exon I of the herpes simplex virus putative terminase subunit, UL15, indicate that the most conserved residues are essential for cleavage and packaging. J. Virol..

[89] Rao V.B., Mitchell M.S. (2001). The N-terminal ATPase site in the large terminase protein gp17 is critically required for DNA packaging in bacteriophage T4. J. Mol. Biol..

[90] Govindaraj R.G., Brylinski M. (2018). Comparative assessment of strategies to identify similar ligand-binding pockets in proteins. BMC Bioinformatics.

[91] Pi F., Zhao Z., Chelikani V., Yoder K., Kvaratskhelia M., Guo P. (2016). Development of Potent Antiviral Drugs Inspired by Viral Hexameric DNA-Packaging Motors with Revolving Mechanism. J. Virol..

[92] Sun S., Rao V.B., Rossmann M.G. (2010). Genome packaging in viruses. Curr. Opin. Struct. Biol..

[93] Waters J.T., Kim H.D., Gumbart J.C., Lu X.J., Harvey S.C. (2016). DNA Scrunching in the Packaging of Viral Genomes. J. Phys. Chem. B..

